# Assessment of immunotoxicity in the 21st century: Where we are and what we need to replace animals

**DOI:** 10.1016/j.cotox.2025.100529

**Published:** 2025-05-28

**Authors:** Victor J. Johnson, Dori R. Germolec, Michael I. Luster, Emanuela Corsini

**Affiliations:** 1 Burleson Research Technologies, Inc., Morrisville, NC, USA; 2 Division of Translational Toxicology, National Institute of Environmental Health Sciences, Research Triangle Park, NC, USA; 3 Laboratory of Toxicology, Department of Pharmacological and Biomolecular Sciences ‘Rodolfo Paoletti’, Università degli Studi di Milano, Milan, Italy

## Abstract

The field of immunotoxicity assessment has traditionally relied on animal models, which have raised ethical concerns and presented limitations in terms of predictive accuracy and relevance to human health. This paper reviews the current state of immunotoxicity evaluation, emphasizing the importance of transitioning away from animal testing. Modern approaches, including in vitro methods, human-based studies, adverse outcome pathways (AOPs), and computational modeling, are discussed as viable alternatives. These non-animal methods offer enhanced predictive power and the potential for regulatory acceptance, though technical and practical challenges remain. Case studies demonstrate the success of non-animal methods, such as AOP-based assessments for skin sensitization and the pyrogen assay using whole blood cytokine assays. Despite these advances, further research is needed in areas like respiratory sensitization, developmental immunotoxicology (DIT), and microphysiological systems (MPS). Recommendations are provided to accelerate the adoption of new approach methodologies (NAMs), focusing on AOP frameworks. In conclusion, the paper highlights the key findings from current non-animal immunotoxicity research and issues a call to action for advancing these methods to improve safety assessment practices in the 21st century.

## Abstract: introduction and historical context of immunotoxicity assessment

The potential for chemicals to modulate immune function, including unintended immune suppression or immune stimulation, is an important concern as immune dysregulation in humans can lead to increasing rates of asthma, allergic contact dermatitis, infectious disease, cancer, and autoimmune disease [1]. Early efforts to assess immunotoxicity relied extensively on animal testing, which included tiered approaches for an integrated assessment of innate, cell-mediated, and humoral immunity where immunosuppression was a concern [2–4], or irritancy, sensitization, or elicitation where hypersensitivity was of concern [5,6]. More recently, the development and validation of alternative approaches to evaluate immunotoxicity has become a priority due to ethical concerns regarding animal welfare and human relevance. The complexity of the immune system makes it unlikely that a single test will be able to assess all the potential adverse effects on immune function. However, promising alternatives have emerged to address immune suppression and sensitization. This review discusses the state-of-the-art, major challenges, and future needs for screening of potential immunotoxic agents using alternative methods.

## Modern approaches to immunotoxicity assessment: new approach methodologies, adverse outcome pathways, and key characteristics

Incredible progress has been made in the direction of developing new methodological approaches in immunotoxicology [7,8]. Spurred by the publication from Organisation for Economic Co-operation and Development (OECD) in 2012 on the adverse outcome pathway (AOP) for skin sensitization (see Modern approaches to immunotoxicity assessment: new approach methods, adverse outcome pathways, and key characteristics below), there has been substantial progress in the use of *in vitro* models to assess chemical sensitization resulting in these methods being accepted to support chemical (including agrochemical formulations) and pharmaceutical registrations by international regulatory agencies. The articulation of the key events that define the AOP for dermal hypersensitivity provided a framework for the development of *in chemico*, *in silico* and *in vitro* techniques that could be used to interrogate the effects of chemicals on each of the key events. Further, the development of defined approaches that evaluate the sensitivity and predictivity of these techniques have shown that the combination of data from multiple alternative methods demonstrated similar or better performance than many of the animal test methods when compared with reliable and reproducible human data [9,10]. This is exemplified by acceptance of numerous new approach methodologies (NAMs) under OECD test guidelines 442C [11], 442D [12], and 442E [13] for skin sensitization hazard identification (see Table 1).

Following an ECVAM workshop in 2003, Corsini and Roggen [14] and Lankveld et al. [15] proposed tiered approaches to *in vitro* screening for immune suppression, starting on myelotoxicity as the first endpoint as immune-related cells develop from pluripotent, hematopoietic stem cells in the bone marrow. Assessment of lymphotoxicity served as a second tier if a compound was not myelotoxic, with function-related assays conducted in a third tier to identify specific targets. The recently adopted OECD test no. 444A: IL-2 Luc assay evaluates immunotoxic effects on T cells by measuring luciferase gene induction for IL-2, IFN-γ, and GAPDH activity [16]. Given the immune system’s complexity, this assay, along with those for skin sensitization, is part of a broader test battery for immunotoxicity assessment. Efforts to develop and standardize NAMs for specific immunotoxic agents continue [7].

The development of NAMs in immunotoxicology has been stimulated, in part, by the identification of AOPs [17]. In 2012, the OECD proposed a formal resource to develop AOPs under the guidance of the Extended Advisory Group on Molecular Screening and Toxicogenomics, which also provides a public available inventory of existing AOPs (https://aopwiki.org/). In AOP Wiki, several AOPs related to immunotoxicity can be found, of which three have been endorsed by the Working Party on Hazard Assessment/Working Group of National Co-ordinators (WNT), namely, ‘Covalent Protein binding leading to Skin Sensitisation’ (ID 40); ‘Inhibition of Calcineurin Activity Leading to Impaired T-Cell Dependent Antibody Response’ (ID 154), and ‘Impaired IL-1R1 signaling leading to Impaired T-Cell Dependent Antibody Response’ (ID 277).

AOPs describing impaired antibody production (AOP IDs 154, 277, 315) are particularly relevant as the T-dependent antibody response (TDAR) is considered important in the assessment of the immunotoxic potential of xenobiotics as it encompasses antigen recognition to T and B lymphocyte signaling, cytokine production, and class switching [3]. The TDAR alone is, however, insufficient because some substances can interfere with innate immunity (e.g. NK cell activity), which the TDAR does not detect.

There are several additional AOPs under development related to immune function and these AOPs will provide a framework to anchor NAMs, facilitating a mechanistic understanding of immune toxicity.

Advancement in computational biology has made it possible for *in silico* methods to offer significant benefits to both regulatory agencies and industry in the assessment of chemical and pharmaceutical safety. At present, the majority of *in silico* models address only skin sensitization, e.g., OECD toolbox, ToxTree, TOPKAT, Derek, and TOPS-MODE [18]. While the models available for predicting respiratory sensitization hold promise [19–21], none of those currently available are accepted for regulatory purposes [22,23] and chemicals with undefined structure, chemical mixtures, and substances containing metals remain a challenge. With the inclusion of artificial intelligence into computational science, *in silico* methods should markedly improve.

Regarding *in silico* models to address immunotoxicity, Banerjee et al. [24] introduced ProTox-II, a free web server designed to predict the toxicity of chemicals (https://tox-new.charite.de/protox_II/). The model leverages molecular similarity, pharmacophores, fragment propensities, and machine learning algorithms to forecast various toxicity endpoints, including immunotoxicity. Another computational model that deserves further investigation is the Universal Immune System Simulator (UISS) [25]. The UISS is a simulation framework designed to model the immune system’s main features and dynamics. It incorporates simulation engines, optimization techniques, and other prediction models using a multiscale, multi-organ, 3D agent-based simulator. Additionally, it includes CrOSSBAR, a module for simulating biological pathways at the molecular level. UISS can replicate various immune responses, including reactions to viruses, bacteria, tumors, autoimmune diseases, and drug-induced effects [26–28]. Such platforms have been used to evaluate the adverse effects of per- and poly-fluoroalkyl substances compounds (PFAS) [29,30]. One of the difficulties in applying *in silico* models to immunosuppression studies is the absence of establishing a clear mode of action. However, the use of reference points such as AOPs and the KCs of immunotoxicants should provide additional information on the biological events underlying immune suppression and allow further development of *in silico* models.

### Key characteristics of immunotoxic compounds

**Figure 1:**
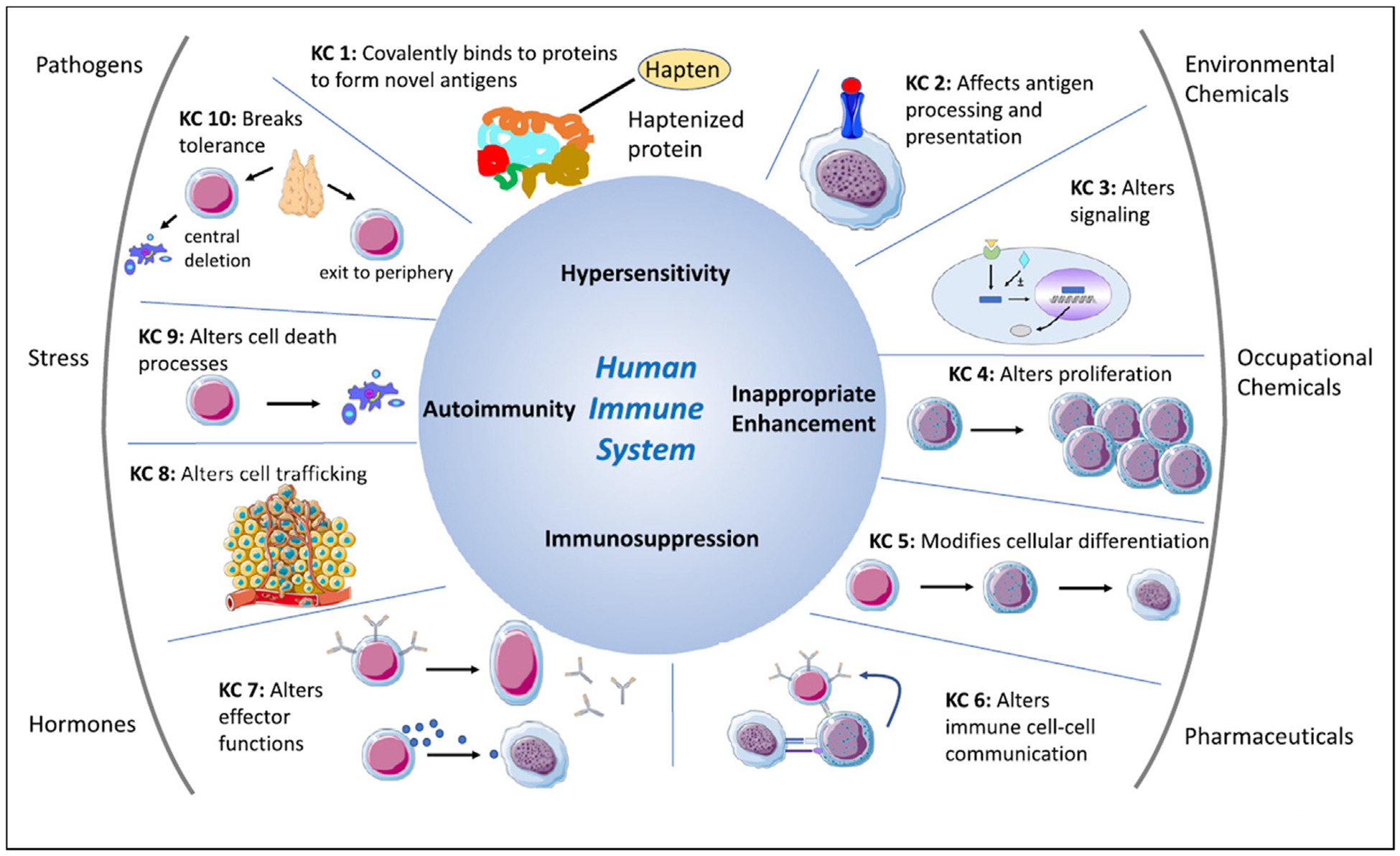
Key characteristics of immunotoxic compounds. The outer ring lists the main categories of compounds that have the potential to influence the immune system. The inner ring illustrates the 10 KCs of immunotoxic agents. Immunotoxic compounds exhibit one or more of the KCs and contribute to immunotoxicity (center) involving hypersensitivity, immune stimulation, immunosuppression, and/or autoimmunity. Reproduced with permission [31].

The key characteristics (KCs), originally developed by the International Agency for Research in Cancer as a framework to assess mechanistic effects of carcinogens, has recently been employed to address potential mechanisms of immunotoxicity [31]. The framework of KCs is valuable for identification of potential immunotoxicants based on exhibiting one or more common modes or mechanisms of action. The KCs for immunotoxic agents include the following: 1) covalently binds to proteins to form novel antigens, 2) affects antigen processing and presentation, 3) alters immune cell signaling, 4) alters immune cell proliferation, 5) modifies cellular differentiation, 6) alters immune cell–cell communication, 7) alters effector function of specific cell types, 8) alters immune cell trafficking, 9) alters cell death processes, and 10) breaks down immune tolerance. The interrelationships between xenobiotic exposures, KCs, and detrimental alterations of immune function are shown in Figure 1. These KCs provide a framework for designing alternative methods to assess immunotoxicity as well as opportunities to identify, characterize, and prioritize testing strategies for compounds that may affect the immune system, with the goal of improving human health risk assessment. As an example, the immunotoxicity KCs provided context for a scoping review of the immunotoxicity of PFAS.

## Advantages and challenges of non-animal methods in hazard identification for risk assessment

Immunotoxicology testing can readily be adapted to incorporate *in vitro* human cell–based assays as alternatives to animal models. Using human primary leukocytes presents some challenges such as their short lifespan and inability to be propagated for extended periods, requiring fresh isolation before use. However, leukocytes can be frozen, and several suppliers provide human peripheral blood mononuclear cells [32]. Donor variability due to genetics (e.g. HLA loci, gene polymorphisms) and epigenetic factors (life-style factors, gender, age), can be significant and must be considered in the experimental design. To mitigate this variability, each donor can serve as their own control. Many of the current *in vitro* techniques lack complex interactions that occur *in vivo* among different cells, tissues, and organs. In the future, immunocompetent microphysiological systems (MPS) may overcome this limitation [33]. Biotransformation is also often a critical issue. For example, low bioavailability due to inadequate metabolism *in vitro* can make some immunosuppressive compounds undetectable or detectable or in the case of sensitization there may be inadequate metabolism of pre-hapten and pro-hapten. One strategy to address this problem is using culture systems with S9 fractions [34,35]. Currently, nominal concentration is often used to define *in vitro* concentration–effect relationships but does not account for differences in bioavailability due to a test material’s reactivity and/or nonspecific binding to media and its constituents [36].

### Regulatory acceptance

Prior to replacing existing current experimental animal tests for regulatory decisions, NAMs require interlaboratory comparability and/or validation. A method is considered validated if its accuracy, reliability, and relevance to an adverse outcome are established using recognized scientific principles [37]. The validation and use of *in vitro* skin sensitization tests as a replacement for existing animal tests, such as the guinea pig maximization test and the local lymph node assay, has been a major accomplishment in the field of immunotoxicology [5,10,11,12,13,38,39]. Skin sensitization is a complex process and the likelihood that a single test can effectively predict human skin sensitizers is unlikely. Realizing this limitation, most approaches have integrated the information from multiple alternative methods developed from *in silico*, *in chemico*, and *in vitro* methods [40–43]. These approaches are aligned to several key events (KEs) involved in the AOP for skin sensitization and include molecular interaction with skin proteins (KE1), inflammatory responses mediated by keratinocytes (KE2), dendritic cell activation (KE3), and T-cell proliferation (KE4). Most approaches have focused on KEs 1, 2, and 3, summarized in Table 1, along with *in silico* computational models (e.g. QSAR Toolbox, Derek Nexus).

OECD (2023a) [10] has published updated test guidelines (TG 497) to identify skin sensitizers using non-animal alternatives that are accepted by many regulatory agencies. The guidelines consist of three rules based defined approaches (DA). The first DA, used for hazard identification, indicates that if any 2 of the 3 tests (i.e. DPRA, KeratinoSens^™^, h-CLAT representing KE1, KE2, KE3, respectively) are positive then the test substance is considered a sensitizer. The second DA represents an integrated testing strategy (ITSv1) based on data from KE1, KE3, and Derek Nexus v6.1.0 along with a fixed data interpretation procedure. The third DA is like the previous DA but uses OECD QSAR Toolbox v4.5 for *in silico* input rather than Derek Nexus. ITSv1 and ITS v2 are also used to discriminate chemicals into three potency categories (category 1A = strong sensitizer; category 1B = other sensitizer, and no categorization (NC = not classified) as based upon the Globally Harmonized System (GHS). These DAs are like those developed by Strickland et al. [43,44], which used DPRA, KeratinoSens, h-CLAT, read-across predictions generated by the QSAR Toolbox and physical properties (e.g. partition coefficients) to identify skin sensitizers. One limitation of these approaches is the lack of predictive points of departure to be used in skin sensitization risk assessment. The approaches using statistical regression, machine learning, and Bayesian models can be employed to derive probabilistic human-relevant PODs, which can be applied to the GHS skin sensitization hazard assessments [41,45]. Models such as the Skin Allergy Risk Assessment Integrated Chemical Environment defined approach which combine NAMs and computational approaches provide quantitative and protective potency estimates that can be used in a regulatory framework [46].

Although U.S. regulatory agencies have yet to established guidelines, most agencies consider non-animal alternative tests useful and sufficient for skin sensitization hazard identification. The Environmental Protection Agency (EPA) Office of Pesticide Programs and Office of Pollution Prevention and Toxics have published a draft interim science policy on the acceptance of alternative approaches for skin sensitization assessments using defined approaches [47]. The National Toxicology Program Interagency Center for the Evaluation of Alternative Toxicological Methods has developed the DASS APP, which applies the defined approaches on skin sensitization (DASS) that are described in OECD TG. 497 and by the U.S. EPA [48]. Together, these efforts demonstrate substantial support for the use of NAMs in immunotoxicity hazard identification for risk assessment [49].

## Future directions and recommendations for NAMs in immunotoxicology

Unlike skin sensitization, NAMs for other sub-disciplines of immunotoxicology including immunosuppression, developmental immunotoxicology, respiratory hypersensitivity, and autoimmunity have not progressed sufficiently to be applicable for risk assessment. Like allergic contact dermatitis, these are also complex biological processes and will likely require an integrated testing approach to successfully conduct hazard ID. Nonetheless, except for autoimmunity, there are numerous assays that, while yet to undergo sufficient validation for regulatory acceptance, show promise as reliable *in vitro* alternatives to animal studies applicable for risk assessment purposes. While there is a continuing need to develop more predictive *in vitro* NAMs, a more urgent need is to identify, adapt, and optimize NAMs already in existence and provide support for test validation. In the following sections, the authors briefly describe some of the more promising NAMs for immunotoxicology testing for risk assessment in key areas.

### Respiratory sensitization

An AOP for respiratory hypersensitivity has been described which proposes that the KEs that contribute to airway hypersensitivity are like those of skin sensitizers [50] including covalent binding of chemicals to proteins, 2) activation of cellular danger signals, 3) dendritic cell migration and activation, and 4) activation, proliferation of T cells. Current data suggest these methods, as described by OECD 442 and 497 guidelines, can successfully identify respiratory sensitizers as well as skin sensitizers, but are unlikely to discriminate between the two [23,51]. Investigators have proposed differences in the AOP that may be leveraged to identify respiratory from skin sensitizers. For example, it has been hypothesized that preferential reactivity to lysine versus cysteine residues on model peptides is a characteristic of chemicals that induce Th2 responses and may be useful for discriminating respiratory sensitizers [50,52,53]. Preferential binding to lysine should be interpreted with caution; however, as a recent evaluation of 200 compounds showed that higher lysine reactivity was more closely associated with chemical structure, acid anhydrides in particular, than representing a unifying characteristic of respiratory sensitizers [54]. Other studies have identified unique genomic signatures following treatment of dendritic cells (i.e. KE3) with respiratory sensitizers not shared with skin sensitizers [55]. A key process for respiratory sensitization is the development of functionally polarized CD4+ type 2 T (Th2) cells in contrast with Th1 cells observed with skin sensitizers. Polarized Th1 and Th2 cells exhibit different functional properties, as well as preferential or selective expression of certain activation markers, cytokines and transcription factors and T-cell proliferation, activation and polarization (KE4) may be used to distinguish skin vs. respiratory sensitization [56]. Investigators using various culture models that mimic the alveolar compartment have identified preferential or selective expression of markers associated with Th2 development that are not detected with dermal sensitizers and/or irritants, such as IL-6, CCL20, GM-CSF, and IL-10, CCL7, CXCL5, as well as OX40L and IL1-R1-1 gene expression [57–61].

Co-cultures that form differentiated epithelial barriers consisting of ciliated cells, mucous secreting cells, and basal cells, together with immune cells are useful for investigating epithelial-immune involvement indicative of respiratory sensitizers. These 3-D models offer the advantage of treatment at the air-liquid interface effectively overcoming water solubility issues expanding the applicability domain [62–64]. A promising co-culture model that is progressing toward validation is the ALIsens assay that leverages cytokine profiles and cell surface activation markers to discriminate respiratory sensitizers [60].

### Systemic immunosuppression

The identification of systemic immunotoxicity had been traditionally conducted in rodents using a battery of tests involving measures of cellular, humoral and innate immunity [3]. These tests rely on interrogating end points that are proximal to the 10 KCs described above and it was recommended that development of NAMs should consider all of them [31]. Fortuitously, *in vitro* tests, using fresh or frozen human peripheral blood cells, have been developed for many of these tests and are commercially available as they are used to identify patients with immunodeficiency diseases and monitor those on immunosuppressive therapies. They include tests for cytokine release, immunophenotyping, NK cell killing, and tests for macrophage and neutrophil activation such as bacterial cell killing and phagocytosis. The most challenging of the methodologies, and arguably the most relevant for immunotoxicology, involve primary cellular and humoral immune responses (i.e. adaptive immunity). Several authors have successfully demonstrated *in vitro* induction of primary antibody responses [65–67] and cytotoxic T lymphocyte activity [68–70] using *in vitro* test systems. The antigens used in these assays tend to be particularly immunogenic, such as hemocyanin, ovalbumin, and TNP conjugates. Investigation of primary, in contrast to secondary, responses *in vitro* can be challenging due to the scarcity of B and/or T cells that recognize a specific antigenic epitope and often require the enhancing effect of alloantigens and/or extended culture periods [71]. To circumvent the need for longer culture periods some investigators have examined secondary responses (a.k.a. recall or memory responses). With these assays, primary exposure occurs *in vivo* and secondary responses are then measured *ex vivo*. It is now relatively common to measure recall responses in human blood cell cultures to viruses such as influenza [72,73] and SARS-CoV-2 [35]. Alternatively, polyclonal stimulators such as Lipopolysaccharide (LPS) or anti-CD3/CD28 antibodies can be used but these inducers bypass some of the early events involved in adaptive immunity [74]. Recently, a human whole blood *in vitro* test system was used to demonstrate that treatment with dexamethasone or benzo(*a*)pyrene resulted in concentration dependent immunosuppression of proinflammatory cytokine production, NK killing activity, and T-cell activation following stimulation with viral antigens [35,35].

### Developmental immunotoxicity testing

To identify the extent of data gaps for developmental immunotoxicity testing and design strategies to address the gaps using NAMs, an international working group was formed in 2019 focusing on alternatives to *in vivo* developmental immunotoxicity testing (https://caat.jhsph.edu/developmental-immunotoxicity/). In addition to tests described above for systemic immunosuppression, the committee identified CD34+ cord blood cells and other human hematopoietic and progenitor stems cells (HPSC) as promising tools for the development of *in vitro* assays focusing on immune cell differentiation and lineage commitment [75]. Utilization of an *in vitro* model using human CD34+ cord blood demonstrated that exposure of the developing culture to 2,3,7,8-tetrachlorodibenzo-p-dioxin impaired B-cell development [76] functioning through aryl hydrocarbon receptor mediated suppression of EBF1 and PAX5 [76]. Most recently, this human CD34+ HPSC model has been shown to recapitulate the development of a near complete spectrum of lymphoid and myeloid lineages, except for T-cells [77]. The addition of Notch ligand was shown to support the development of the T-cell lineages from CD34+ HPSC cultures *in vitro* [78]. Defining ‘normal’ for key processes at all stages of immune development including, but not limited to, cell differentiation, lineage commitment, proliferation, migration, and function will facilitate identification of those processes relevant to immunotoxicity safety assessment and strategic development of NAMs targeting key events.

## Conclusions

As the field of immunotoxicology progresses toward increasing adoption of NAMs, it is important to understand that they can offer several advantages over *in vivo* methods, including mechanistic understanding, easier species extrapolation, and the reduction, refinement, and replacement of animal use. They also present some limitations such as for discriminating potency, limits to applicability domain, as well as limitations regarding chemical biotransformation and bioavailability. Currently most of the successes in the implementation of these assays in immunotoxicology have involved cell lines. However, considerable efforts are underway to use more complex culture systems with primary human cells.

## Figures and Tables

**Table 1:** OECD test method guidelines for hazard identification of skin sensitizers.

OECD 442C	KE1	Covalent binding of electrophilic substances to nucleophilic centers on skin protein	Direct peptide reactivity assay (DPRA), amino acid derivative reactivity assay (ADRA), kinetic direct peptide reactivity assay (kDPRA^a^).
OECD 442C	KE2	Keratinocyte inflammation using luminescence detection to measure gene expression of antioxidant/electrophile response element (ARE)-dependent pathways.	ARE-Nrf2 luciferase KeratinoSens^™^ test method, ARE-Nrf2 luciferase LuSens test method. 1.1.3. Key event three—activation of d
OECD 442E	KE3	Dendritic cell activation	Expression of cell surface markers CD86 and CD54 by human Cell Line Activation Test (h-CLAT), expression of CD86 using U937 cell line activation test (U-SENSTM), IL-8 Gene Assay (IL-8 Luc Assay) that measures IL-8 mRNA.

KE, key event.

a For identification of strong sensitizers.

## Data Availability

No data was used for the research described in the article.
